# OSCAR-Assessing Individual Risk Profiles of MRONJ Patients

**DOI:** 10.3390/clinpract16050094

**Published:** 2026-05-19

**Authors:** Felix Pitka, Florian Böhrnsen

**Affiliations:** 1 Clinic for Maxillofacial Surgery, University Medical Center Goettingen, 37075 Goettingen, Germany; 2 Department of Maxillo-Facial-Surgery, Medical University of Luebeck, 23562 Luebeck, Germany

**Keywords:** MRONJ, antiresorptive therapy, bisphosphonates, Denosumab, clinical scoring system

## Abstract

Objective: To develop an individual assessment for patients suffering from medication-related osteonecrosis of the jaw (MRONJ), we developed a scoring system that integrates anamnestic, clinical, and radiological parameters to facilitate the process of therapeutic decisions during MRONJ therapy. Methods: In this study, clinical data as well as diagnostic CT scans from 41 MRONJ patients undergoing antiresorptive therapy were analyzed to develop an Osteonecrosis Scoring Clinical Assessment and Radiological Report (OSCAR). Results: Total OSCAR scores ranged from 4 to 30, with lower scores demonstrating less severe disease progression following a de-escalated therapy. OSCAR scores above 15 were associated with higher bone density and an increased need for surgical intervention, with 70% of Denosumab and 71% of bisphosphonate patients requiring surgical intervention. Conclusions: Patients with an OSCAR below 12 were unlikely to require multiple surgical interventions. Since OSCAR parameters are routinely collected during hospitalization, this opens the possibility for future AI-assisted patient assessment and treatment planning.

## 1. Introduction

Bisphosphonate (BP) and Denosumab (DB) are antiresorptive agents widely used in patients with pathologic bone resorption due to osseous metastasis of solid tumors, osteoporosis, multiple myeloma, or Morbus Paget [1,2]. The primary effect used during antiresorptive therapy is the reduction in physiological bone remodeling by inhibiting osteoclast activity. BP and DB differ in targets; pharmacokinetics and pharmacodynamics with BP express a half-life of up to 10 years, while DB demonstrates a half-life of around 26 days [1,2]. Both lead to a significant increase in total bone mineral density [3,4,5,6]. However, the occurrence of site-specific negative effects is similar [2,6]. The most feared associated complication is the medication-related osteonecrosis of the jaw (MRONJ) [1,2,7,8,9,10]. The multifactorial pathogenesis of MRONJ remains incomplete [1,2,9]. A key pathophysiological process appears to be reduced blood supply and bone remodeling due to inhibition of regenerative cellular activity [1,2,9,11]. The estimated cumulative incidence of MRONJ in patients receiving BP intravenously for malignant diseases varies between 0.8% and 15% [1,2,7,8,12]. In contrast, the incidence in patients receiving oral antiresorptive therapy (i.e., osteoporosis) is significantly lower at approximately 0.01% to 0.1% [13]. Successful treatment of MRONJ varies significantly and often requires surgical intervention [11,14,15]. Surgical treatment of oral pathologies and MRONJ, however, may increase the risk of triggering additional osteonecrotic complications requiring secondary interventions [11,15,16,17]. Therefore, a comprehensive scoring and clinical assessment of patients is imperative to improve MRONJ treatment and outcome [15]. Central contributors to a scoring system are individual risk factors, medical illness, type of antiresorptive medication, and osseous parameters [9]. While established systems such as AAOMS [13] rely primarily on clinical presentation, there are only a limited number of scoring systems that include osseous parameters, evaluated via standardized technical examination [15]. Although dual-energy X-ray absorptiometry (DXA) is the most widely accepted technique for measurements of bone mineral density (BMD), it is not routinely performed. Hamada et al. demonstrated the value of CT analysis to detect early stages of MRONJ using easily accessible and internationally accepted Hounsfield Units (HU) as a predictive parameter for MRONJ [18]. The goal of this study was to create an individual risk profile for patients taking antiresorptive medications by utilizing a scoring system that combines anamnestic, clinical, and radiological parameters to optimize the assessment of patients suffering from MRONJ.

## 2. Materials and Methods

### 2.1. Study Design

This study was made in accordance with the ethical guidelines of the revised declaration of the World Medical Association of Helsinki. Ethical approval was obtained from the ethical review committee of University Medicine Göttingen (No. 11/8/20, date: 28 October 2025). We analyzed 41 (18 female, 23 male) patients suffering from MRONJ, treated at the Department of Oral and Maxillofacial Surgery at the University Medicine Göttingen between 2015 and 2020. Patients received either BP (*n* = 21) or DB (*n* = 20) treatments. All patients were diagnosed with MRONJ based on the diagnostic criteria of the American Association of Oral and Maxillofacial Surgeons [2]. Clinical data and routinely performed head and neck CT scans of patients suffering from MRONJ lesions of the mandible were analyzed and internally referenced. Furthermore, CT scans of 17 patients treated for jaw fractures with no additional jaw pathologies were analyzed for reference. Inclusion criteria for BP and DB patients included treatment with intravenous, subcutaneous, or oral antiresorptive medication. To compensate for misinterpretation, patients who received both antiresorptive substances (BP + DB) were excluded from this study. Patients in the reference group who had undergone radiation, antiresorptive, chemotherapeutic treatment, and/or were suffering from bone pathologies, as well as taking medication that affected bone metabolism, and cases where MRONJ had spread ubiquitously throughout the jawbone, were excluded.

### 2.2. CT Data and Statistical Analysis

**Figure 1:**
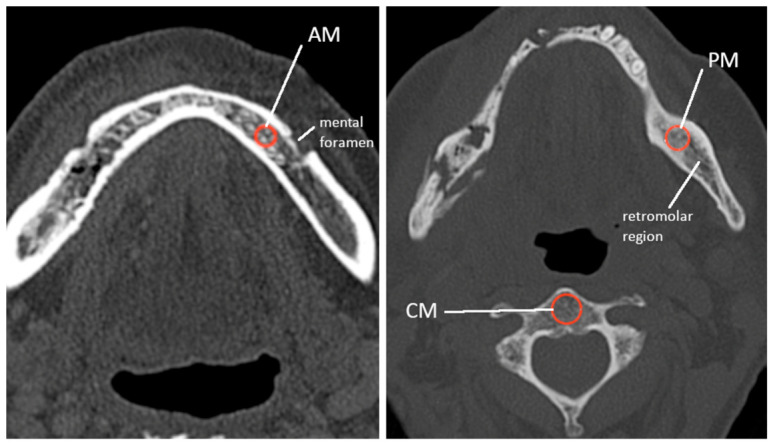
Locations of CT bone density measurements in the mandible and the 2nd vertebra. AM: anterior mandible; PM: posterior mandible; CM: control.

All routine CT scans were performed on a SOMATOM Definition AS+ (Siemens AG, Healthcare Sector, Erlangen, Germany) by the Institute for Diagnostic and Interventional Neuroradiology at University Medicine Göttingen. Images were imported using the reporting software syngo.via VB40A (Siemens Healthineers, Erlangen, Germany) and measured on a standard radiologic workstation. Parallel series were reconstructed with a slice thickness of 0.5 mm and a distance between the images of 0.5 mm. The field of view (FoV) was specified to 230 × 230 mm. Resulting serial reconstructions were aligned to the occlusal plane. Analysis was performed contralaterally of the necrotic lesion, 1: anterior mandible, preforaminal (AM), and 2: posterior mandible, postforaminal (PM). The internal reference control (CM) was defined outside of the affected oral cavity within the body of the second cervical vertebra (Figure 1). All measurements were described in Hounsfield Units (HU). For each measurement, a circular region of interest (ROI) was placed in cancellous bone, avoiding measuring in compact bone. All BMD measurements were electronically registered and evaluated using Microsoft Excel (Version 16.64; Microsoft Corporation, Redmond, WA, USA). Measurements were analyzed by calculating the median, mean, and corresponding standard deviations of the defined ROIs and comparing them, respectively. Group differences were tested using unpaired t-tests under the assumption of unequal variances (Welch’s *t*-test), with a *p*-value of ≤0.05 considered statistically significant.

### 2.3. OSCAR—Osteonecrosis Scoring Clinical Assessment and Radiological Reports

OSCAR is composed of three categories: anamnestic parameters [1], clinical parameters [2], and radiological bone density reports [3]. Selection of parameters, weighting, and scoring was based on comprehensive reviews of the existing literature [7,14,19,20,21,22]. A detailed description of the OSCAR parameters is given in Table 1. The anamnestic parameters are comorbidity (C), age (A), previous operation (O), and multiple operations (MO) of MRONJ lesions. The clinical parameters include lesions of the mandible (L) and disease-dependent antiresorptive application form and interval (DAFI). CRP and leukocyte (LC) levels were determined prior to operative therapy. To ensure a representative assessment of the individual bone architecture and to minimize the influence of local anatomical variations or imaging artifacts, BMD parameters were measured in standardized AM, PM, and CM locations. All measurements were compared to each other within AM, PM, and CM locations as well as BP and DB groups, respectively. Subsequently, HU values were analyzed by calculating the median and classifying them into quartile ranges (Q1, Q2, Q3, Q4) for AM, PM, and CM locations. Like the Agatston score [23], it was determined whether an individual value was above or below Q4, respectively. Based on this classification, AM, PM, and CM were assigned an OSCAR value of 0 (Q1–Q3) or 2 (Q4), reflecting the OSCAR’s systemic objective rather than focusing on isolated local density changes. Additional OSCAR parameters were also assigned values of 0, 1, 2, or 3 (Table 1), and the OSCAR calculated

OSCAR = C × 1 + A × 1 + O × 2 + MO × 4 + L × 2 + DAFI × 2 + CRP × 1 + LC × 1 + AM × 3 + PM × 3 + CM × 1

Abbreviations: C: comorbidity; A: age; O: operation; MO: multiple operations; L: lesion of the mandible; DAFI: disease-dependent antiresorptive application form and interval; CRP: C-reactive protein; LC: leukocyte; AM, PM, CM: area of measured BMD.

## 3. Results

### 3.1. Primary Diseases of MRONJ Patients in BP and DB Groups

BP-treated patients showed progressive osteoporosis in 33% of cases, while 67% were suffering from bone degradation due to malignant disease. In the DB group, progressive osteoporosis was found in 15% of patients, while 85% had tumor-related bone degeneration. The distribution of specific primary diseases is detailed in Table 2.

### 3.2. Dosage and Application of Antiresorptive Medication in MRONJ Patients

Detailed medication, dosage, and application form of antiresorptive treatment protocols analyzed are shown in Table 3.

### 3.3. Disease-Dependent Antiresorptive Application Form and Interval (DAFI)

The distribution of patients in the BP and DB groups, categorized via primary disease in correlation to the form of drug application as well as intervals of antiresorptive medication, is shown in Table 4. Patients in the BP group predominantly received intravenous treatment for malignant diseases (67%). DB patients suffering from osteoporosis received subcutaneous administration of Denosumab twice a year (15%). Patients treated with malignant diseases received subcutaneous DB applications once a month (85%).

### 3.4. Surgical Interventions and Elevated Bone Density in BP and DB Groups

**Figure 2:**
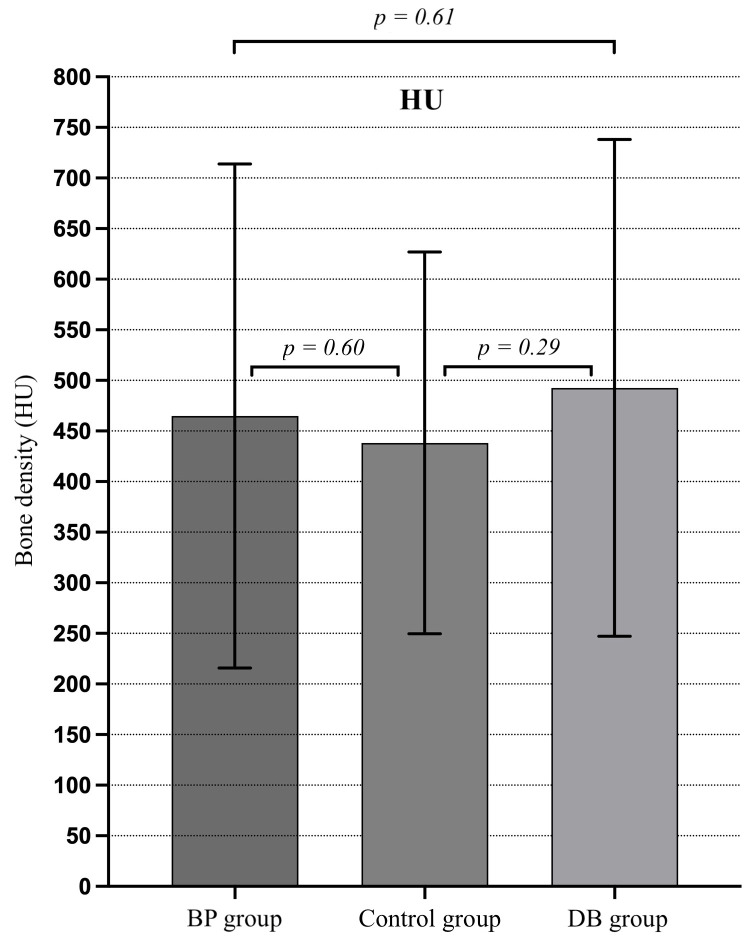
HU comparison of bisphosphonate (BP), Denosumab (DB), and the control group demonstrates no significant intergroup differences.

In 33% of cases with oral administration of BP, surgical intervention was necessary. In addition, surgical intervention was required in 87% of cases with i.v. BP administration. Overall, 70% of the DB patients and 71% of the BP patients required surgical intervention. Respectively, 29% of patients in the BP and 30% in the DB group had no immediate need for operative therapy and were treated conservatively. However, 38% of patients in the BP group who received surgical treatment had to undergo multiple procedures, compared to 10% in the DB group. BMD in BP patients at AM, PM, and CM ROI ranged from 118 to 1083 HU. In comparison, DB patients showed HU measurements from 128 to 1072, respectively. Our findings demonstrated AM and PM bone density variation in accordance with the number of decortications performed. The median bone density in the anterior mandible for patients who did not undergo surgery was 372.5 HU. Notably, the median bone density increased in both groups who underwent surgical intervention (one surgery, 452 HU/multiple surgeries, 509.25 HU). Since HU values did not meet normal distribution, BMD OSCAR scoring was based on median and quartile classification [23]. Patients were classified using quartile groups based on the distribution of their AM, PM, and CM measurements, respectively. The first quartile (Q1) showed patients with AM, PM, and CM bone density values below 346 HU (*n* = 11). The second quartile (Q2) showed values from 347 to 467.5 HU (*n* = 10), and patients in the third quartile (Q3) demonstrated values from 468 to 756 HU (*n* = 9). In total, 11 patients expressed values above 756.375 HU and were grouped in the fourth quartile (Q4). AM and PM measurements were combined into a single DB and BP patient profile, respectively, and compared to an external reference control (*n* = 116). We observed no significant difference in BMD comparing BP (*p* = 0.60), DB (*p* = 0.29), and control groups, as well as intergroup (DB/BP *p* = 0.61) measurements. However, mean HU values of DB and BP demonstrated a trend towards higher bone density levels when compared to an external reference control (Figure 2).

**Figure 3:**
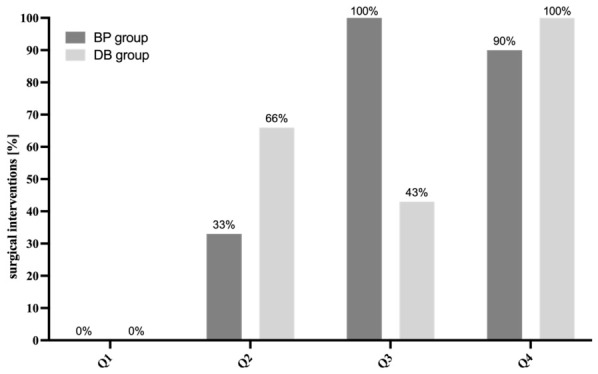
DB and BP patients showed increasing surgical interventions within the 3rd and 4th BMD quartiles (Q3, Q4).

None of the BP patients with bone density values within the first quartile underwent surgical treatment. However, the number of surgical interventions increased to more than 90% in the third and fourth quartiles in patients treated with BP. (Figure 3). Comparably, none of the DB patients with bone density values within the first quartile underwent surgical treatment. DB patients showed a mild increase in surgical interventions in the second and third quartiles but a surge of operative therapy above 90% in the fourth quartile compared to BP patients.

### 3.5. Age and Comorbidity of MRONJ Patients

At the time of CT scanning, patients’ mean age was 71.5 ± 11.9 (SD) years in the BP group and 77.9 ± 6.3 (SD) years in the DB group. Age parameters were categorized into two groups (≤69 and ≥70 years) based on the mean age of our entire study group. Most patients suffered from an average of three comorbidities. Table 5 shows the distribution of MRONJ patients’ comorbidities in the BP group and the DB group. Medical records were recorded at the time of the first consultations.

### 3.6. CRP and Leukocyte Levels of Patients in BP and DB Groups

**Figure 4:**
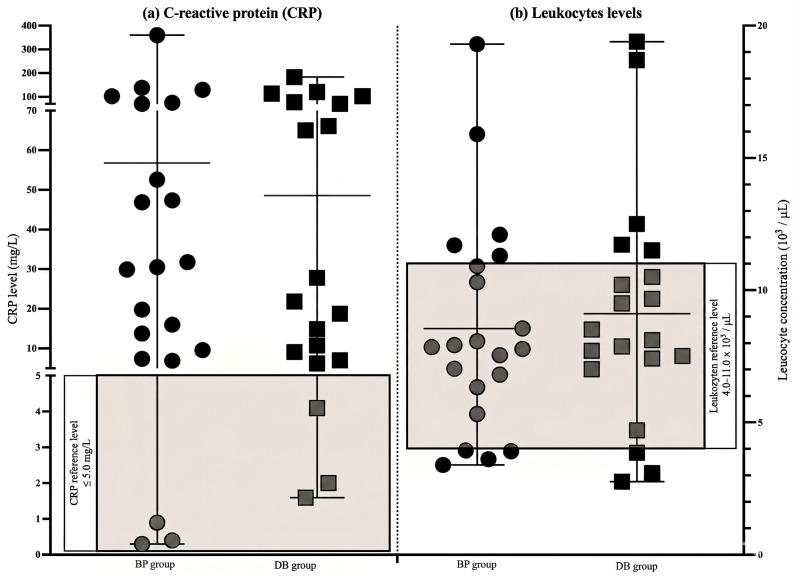
CRP (**a**) and leukocyte levels (**b**) in patients undergoing BP and DB therapy. In total, 86% of patients in the BP and 80% in the DB group showed elevated CRP values above reference levels indicated in boxgraphs.

In total, 86% of patients in the BP and 80% in the DB group showed elevated CRP values of ≥5.0 mg/L (Figure 4). Analyzing leukocyte values, 33% of patients in the BP group and 40% of DB patients were either below 4.0 × 10^3^/µL or above 11.0 × 10^3^/µL, placing them outside the normal range (4.0–11.0 × 10^3^/µL). Patients outside the range were attributed an OSCAR score value of 1.

### 3.7. OSCAR Scoring of Patients Receiving BP and DB Therapy

**Figure 5:**
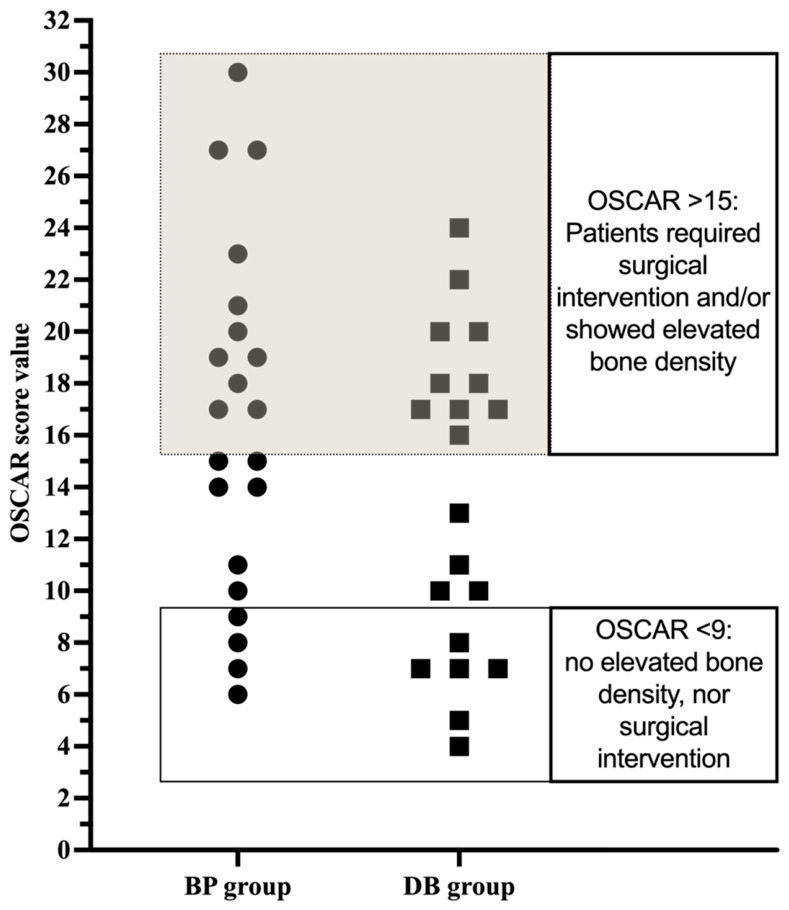
OSCAR score of patients in the BP group and the DB group. Patients who scored above 15 in OSCAR demonstrated elevated bone densities and often required more than one surgical intervention.

Total OSCAR score values ranged from 6 to 30 points in the BP group and from 4 to 24 points in the DB group (Figure 5). In patients exhibiting an OSCAR score below 9, no increased bone density was found, nor did they require surgical intervention in either study group. The data revealed that in patients of both study groups, OSCAR values above 12 were associated with an increased mean bone density. Patients in the BP group often required more than one operative treatment when scored above 15. In comparison, an OSCAR threshold of 16 was evident in the DB group when surgical therapy was necessary.

## 4. Discussion

The primary objective of this study was to develop a tool for individual disease and treatment assessment of MRONJ patients. While established systems like AAOMS [13] rely primarily on clinical presentation, there is only a limited number of scoring systems that include osseous parameters, evaluated via standardized technical examination [15]. Although no significant BMD differences were observed comparing BP, DB, and control groups, measurements were consistent with the previous literature [19], suggesting that in addition to clinical parameters, bone architecture is vital to the pathophysiology of MRONJ [13,19,24]. Evaluating BMD using diagnostic CT scans represents a highly efficient approach, as these scans are routinely available and do not require additional DXA radiation exposure [18]. Notably, our study demonstrated that 90% of BP patients and 100% of DB patients evaluated within the highest quartile (Q4) required surgical intervention and are likely to undergo multiple procedures. This stresses the importance of BMD measurement for identifying patients via OSCAR evaluation. In addition, OSCAR takes the following parameters into account: comorbidity (C), age (A), CRP and leukocytes (LCs), disease-dependent antiresorptive application form and interval (DAFI), operative therapy (O), multiple operations (MO), and lesions of the jaw (L). While all parameters contribute to the OSCAR, DAFI, O, L, AM, PM, CM, and MO have shown a higher correlation to disease progression, resulting in a higher score weighting [1,2,11,14,16,20,25].

Since previous studies have shown that the type and duration of antiresorptive therapy demonstrate a significant influence on MRONJ progression, DAFI was factored in at a higher impact when assessing the OSCAR score [1,2]. This is of importance since prolonged exposure to antiresorptive drugs increases the risk of MRONJ. However, the exposure-time-dependent onset of MRONJ may vary and the development of additional comorbidities may influence bone metabolism over time [1,2,7,12,25]. To mitigate this limitation, comorbidities were included in addition to the DAFI when evaluating the OSCAR score. Furthermore, patients receiving antiresorptive drugs as part of tumor therapy show a higher risk for MRONJ over time due to the combination of DAFI and the underlying disease [1,2,25].

In this study, we observed that the need for surgical decortication varied significantly depending on the method of antiresorptive drug administration. In total, 87% of patients receiving i.v. BPs required surgical decortication compared to 33% with p.o. bisphosphonates. Since our study showed a correlation between bone density and the need for surgical intervention in MRONJ patients, these two parameters were weighted higher in the OSCAR score. Previous studies also report correlations between increased bone density and an increased risk of frequent operative therapy [9,19]. This underlines the importance of BMD for risk evaluation, therapy, and outcome monitoring in patients suffering from MRONJ [15,18]. In our study, 90% of BP and 100% of DB patients with HU values in the fourth quartile underwent at least one surgical decortication procedure, while patients with lower HU values, especially within the first quartile, had the lowest need for operative treatment. Patients within the second quartile showed a considerably higher risk profile when treated with DB in comparison to patients receiving BP (66% vs. 33%). Our data stresses that DAFI, BMD, and antiresorptive treatment are important factors in monitoring successful MRONJ therapy [19,24]. Surgical intervention increases the risk of MRONJ progression, especially when undergoing multiple procedures [17,21,26]. Our study demonstrated that 38% of patients in the BP group underwent multiple operations, increasing the weight of MO in OSCAR evaluation. It is known that initial necrotic lesions often progress to advanced stages of MRONJ [16]. Our data shows that 71% (BP group) and 70% (DB group) of patients suffering from recurring lesions often required surgical intervention. This highlights the significant risk of exacerbated disease and stresses its weight in OSCAR scoring [7,17]. MRONJ correlates with inflammatory progression represented via CRP and leukocyte expression. Studies have shown that MRONJ patients often present elevated CRP and leukocyte levels [17,21,26], indicating an inflammatory response associated with an increased risk of recurrent and prolonged disease [21,26]. In our study, 80% of patients in the DB group and 86% of patients in the BP group demonstrated elevated CRP and leukocyte levels. Previous studies have identified age as a relevant factor influencing both the development and severity of MRONJ. The bone turnover rate decreases significantly starting at the age of 70 [9,27]. In addition, the prevalence of comorbidities increases with advancing age [1,9]. The selection of 70 years as a cut-off value in the OSCAR score is supported by the demographic characteristics of our study group, where the mean age was 71.5 (BP) and 77.9 years (DB). However, we acknowledge that other demographics and health systems may require adaptations of the age threshold and risk profiles.

Despite advances in understanding MRONJ, there are only a few scoring systems available that take clinical and radiological bone density parameters into consideration [15]. Today, there is still neither a defined treatment protocol [1,14,28] nor a comparability scoring of clinical outcomes, stressing the need for a better understanding of MRONJ therapy and monitoring. The goal of OSCAR is to improve the assessment of individual risk, progression, therapy, and clinical outcome profiles of MRONJ patients undergoing antiresorptive therapy, allowing for optimized interdisciplinary communication. Our data showed that patients with an individual OSCAR score below 9 did not show increased bone density, nor did they require surgical intervention. However, patients with OSCAR values above 15 often required several surgical interventions. Moreover, we observed an increased BMD in both groups. Employing OSCAR scoring, it can be assumed that patients with a score lower than 12 are unlikely to require multiple surgeries in the future and could be monitored accordingly. However, we acknowledge that our results are limited. Due to the relatively small sample size and limitation to MRONJ lesions of the mandible, we refrain from overinterpretation. The current OSCAR should not be considered finite. While our results are promising, further validation in prospective multicenter studies and different health and social situations is required to confirm its clinical reliability. Additional factors could also encompass dosage and administration changes for patients undergoing long-term therapy or who are experiencing changes in medication due to adverse side effects. Nevertheless, our study opens the possibility for AI-assisted models to use OSCAR for risk and therapy assessment of patients, lowering the barrier for interdisciplinary communication and successful MRONJ treatment in the future.

## 5. Conclusions

The OSCAR score provides a new individual risk assessment for patients suffering from medication-related osteonecrosis of the jaw (MRONJ). OSCAR uniquely combines clinical and anamnestic parameters with radiological bone mineral density measurements. While still preliminary, OSCAR suggests that a score below 12 indicates a low risk profile for severe disease progression, whereas scores above 15 may identify high-risk patients. Future studies should focus on multicenter validation to establish OSCAR as a robust standardized tool for MRONJ risk assessment.

## Figures and Tables

**Table 1:** Parameters used in the Osteonecrosis Scoring Clinical Assessment and Radiological Report (OSCAR).

Parameters	Criteria	Points
Comorbidity	Patient has no pre-existing general disease Patient has a pre-existing general disease	0
		1
Age	Patients age ≤ 69 years Patients age ≥ 70 years	1
Previous operation Multiple operations	MRONJ has not been subject to surgical intervention MRONJ has undergone 1 surgical intervention MRONJ has undergone >1 surgical intervention	1 2
Lesion of the mandible	AAOMS-classified lesion—non-operative therapy AAOMS-classified lesion—operative therapy	1
Disease-dependent antiresorptive application form and interval (DAFI)	Diagnosis-correlated interval and application form (i.v. p.o., s.c. *) osteoporosis + s.c (2×/year) osteoporosis + p.o. (1×/week) osteoporosis + i.v. (1×/month) malignant disease + s.c. (1×/month) malignant disease + p.o. (1×/week) malignant disease + i.v. (1×/month)	1 2 2 2 2 3
C-reactive protein (CRP)	CRP level within the clinical norm (≤5.0 mg/L) CRP level above 5.0 mg/L	1
Leukocytes	Leukocyte level between 4.0 and 11.0 × 10^3^/µL Leukocyte level below 4.0 or above 11.0 × 10^3^/µL	1
Bone density **	Bone density values within the 1st to 3rd quartile (Q1–Q3) Bone density values within the 4th quartile (Q4)	2

* Application form: i.v. = intravenously, p.o. = per os, s.c. = subcutan. ** Bone density values were evaluated within the localizations AM, PM, and CM and grouped into quartiles, respectively. Assigned points were used in the OSCAR calculation.

**Table 2:** Primary disease of MRONJ patients undergoing BP or DB therapy.

Primary Disease	BP Patients (%)	DB Patients (%)
Osteoporosis	33%	15%
Breast cancer	28%	10%
Prostate cancer	10%	45%
Renal cancer	14%	15%
Multiple myeloma	10%	10%
Solitary plasmacytoma	5%	-
Squamous cell carcinoma	-	5%

**Table 3:** Antiresorptive medication, application, dosage, and interval protocols of MRONJ patients.

Group	Medication	Application Form	Dosage	Interval	Patients %
Bisphosphonates	Zoledronate	i.v.	4 mg	1×/month	29%
	Pamidronat	i.v.	90 mg	1×/month	7%
	Alendronate	p.o.	70 mg	1×/week	15%
Denosumab	Prolia ^a^	s.c.	60 mg	2×/year	37%
	XGEVA ^b^	s.c.	120 mg	1×/month	12%

^ab^ Denosumab (Amgen Inc., Thousand Oaks, CA, USA).

**Table 4:** MRONJ patients’ disease-dependent antiresorptive application form (DAFI).

Primary Disease + Application (Interval)	BP Patients (%)	DB Patients (%)
Osteoporosis + s.c. (2×/year)	0%	15%
Osteoporosis + p.o. (1×/week)	28.5%	-
Osteoporosis + i.v. (1×/month)	4.5%	-
Malignant disease + s.c. (1×/month)	0%	85%
Malignant disease + p.o. (1×/week)	0%	-
Malignant disease + i.v. (1×/month)	67%	-

**Table 5:** Comorbidities of MRONJ patients in the BP and DB groups.

Comorbidity	BP Patients %	DB Patients%
Cardiovascular (hypertension, coronary heart disease, arteriosclerosis, deep venous thrombosis)	67%	45%
Endocrine (diabetes mel., hyperthyroidism, hypothyroidism)	24%	30%
Renal (renal failure, hydronephrosis)	10%	15%
Other	24%	10%
None	10%	5%

## Data Availability

The data presented in this study are available on request from the corresponding author due to institutional and EHDS regulations.

## Abbreviations

The following abbreviations are used in this manuscript:

MRONJ	Medication-Related Osteonecrosis of the Jaw
OSCAR	Osteonecrosis Scoring Clinical Assessment and Radiological Report
BP	Bisphosphonates
DB	Denosumab
CT	Computed Tomography
AI	Artificial Intelligence
DXA	Dual-Energy X-ray Absorptiometry
BMD	Bone Mineral Density
HU	Hounsfield Units
ROI	Region of Interest
FoV	Field of View
AM	Anterior Mandible
PM	Posterior Mandible
CM	Control Measurement (second cervical vertebra—Axis)
DAFI	Disease-dependent Antiresorptive Application Form and Interval
CRP	C-reactive Protein
LC	Leukocyte
SD	Standard Deviation
Q1–4	First to Fourth Quartile
i.v.	Intravenous
p.o.	Per os (oral administration)
s.c.	Subcutaneous
OP/pre-OP	Operation/preoperative
PEC	Squamous Cell Carcinoma
